# Acquired Tracheomalacia Following Tracheostomy: A Case Report and Literature Review

**DOI:** 10.7759/cureus.83350

**Published:** 2025-05-02

**Authors:** Ramli Farid Syamil, Mawaddah Azman

**Affiliations:** 1 Otolaryngology - Head and Neck Surgery, Universiti Kebangsaan Malaysia, Kuala Lumpur, MYS

**Keywords:** airway collapse, case report, tracheomalacia, tracheostomy, tracheostomy complications

## Abstract

Tracheostomy is a widely performed procedure to manage prolonged respiratory failure, but it is not without risk. One rare but serious late complication is acquired tracheomalacia, which can lead to airway collapse and respiratory distress. We report the case of an 82-year-old male patient diagnosed with acute inflammatory demyelinating polyneuropathy who underwent a tracheostomy after failed extubation. The procedure was uncomplicated, and the patient was successfully decannulated after a few months. However, he developed biphasic stridor two months following decannulation. Further evaluation revealed a focal collapse of the right lateral tracheal wall, consistent with tracheomalacia. Given the patient’s stable condition and localized collapse, a conservative management approach using continuous positive airway pressure (CPAP) was adopted, resulting in notable symptomatic improvement. This case highlights the importance of considering tracheomalacia in patients with prolonged intubation or challenging decannulation. Timely endoscopic evaluation is essential for diagnosis, and conservative measures like CPAP may be effective in selected cases, potentially avoiding the need for surgical intervention. The report emphasizes the need for clinical vigilance in the post-tracheostomy period and supports the role of individualized management strategies for late-onset airway complications.

## Introduction

Tracheostomy is among the oldest documented surgical procedures, with evidence of its practice dating back to 3600 B.C. in ancient Egypt [1]. This procedure involves creating an opening in the anterior trachea to establish an alternative airway, thereby assisting respiration. In modern medicine, tracheostomy is commonly employed to aid in weaning patients off mechanical ventilation, which can help optimize the utilization of intensive care unit (ICU) resources and increase bed availability.

Although tracheostomy is now commonly performed, the complication rate varies widely, from as low as 2.1% up to 20% [2]. Early complications of tracheostomy can range from hemorrhage, misplacement of the tracheostomy tube into a false passage, and surgical damage to surrounding structures. Late complications may include milder conditions such as transient tracheitis and stoma cellulitis, as well as more severe, potentially life-threatening issues such as subcutaneous emphysema, pneumothorax, tracheal stenosis, and tracheomalacia [3,4].

Tracheomalacia, though rare, is a significant complication of tracheostomy that can lead to respiratory distress. Despite its uncommon occurrence, its impact on patient outcomes makes it an important consideration in tracheostomy care. In this case report, we present an unusual instance of tracheomalacia that developed following a tracheostomy.

## Case presentation

An 82-year-old male patient initially presented with a two-day history of bilateral upper and lower limb weakness. On the second day of admission, he developed respiratory distress necessitating endotracheal intubation. A diagnosis of acute inflammatory demyelinating polyneuropathy was established. After 13 days of mechanical ventilation and failed extubation attempts, he was referred to our team for tracheostomy due to prolonged ventilatory support.

The tracheostomy was performed without complications using a horizontal incision between the second and third tracheal rings, and a size 8 single-lumen cuffed tracheostomy tube was inserted. Postoperatively, the patient continued ventilator support in the general ward until he was successfully weaned to room air. On postoperative day seven, the tracheostomy tube was changed to a double-lumen type, and the patient was discharged after 40 days of hospitalization.

After a successful one-month trial of a spigot at home, the patient was electively admitted for tracheostomy decannulation. Pre-decannulation endoscopic evaluation revealed minimal suprastomal granulation with no evidence of tracheomalacia. However, on the first day post-decannulation, the patient developed biphasic stridor with significant airway compromise. He underwent urgent laryngobronchoscopy, which revealed suprasternal granulation tissue, which was subsequently removed. Further examination identified a stenotic segment and granulation tissue in the subglottic region. The stenosis was treated with CO₂ laser incision, and the granulation tissue was excised using cup forceps. Postoperatively, the patient was able to breathe spontaneously on room air and was discharged with a planned outpatient follow-up.

**Figure 1:**
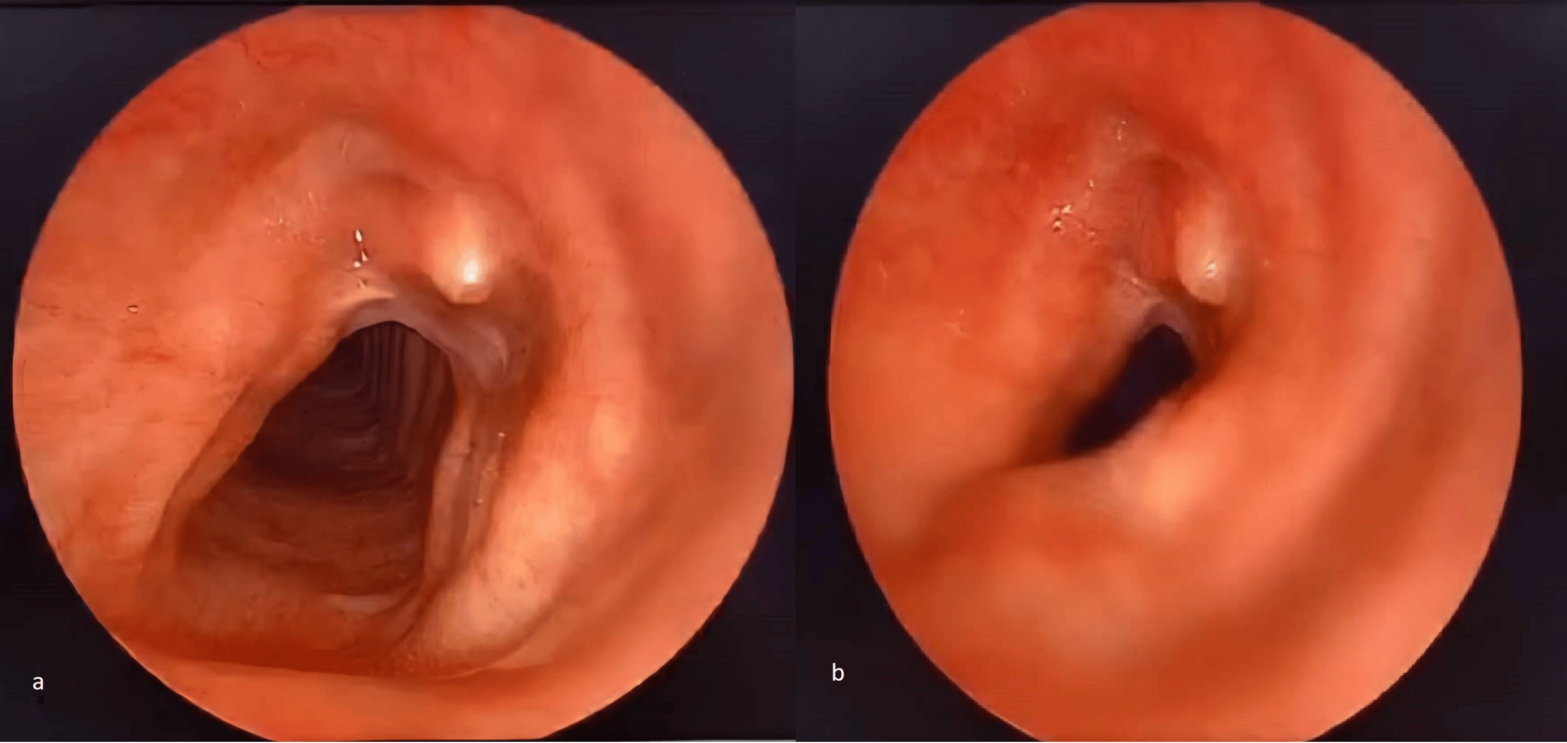
Endoscopic view of the trachea during direct laryngoscopy (a) No collapse of the tracheal wall structure before jet ventilation; (b) focal right lateral tracheal wall collapse upon jet ventilation

Two months later, he developed exertional noisy breathing and chose to be readmitted for further evaluation. An elective suspension laryngoscopy was performed, revealing focal collapse of the right lateral tracheal wall (Figure 1), a feature of tracheomalacia. Postoperatively, a discussion was held with the patient, and a decision was made to proceed with conservative management. The patient was discharged three days later and is currently using continuous positive airway pressure (CPAP) as part of his treatment plan.

## Discussion

Tracheostomy is a commonly performed procedure in critically ill patients requiring prolonged mechanical ventilation for acute respiratory failure and airway issues. While it is an indispensable life-saving intervention, it is often associated with complications. Complications of tracheostomy are categorized based on the time interval from the procedure to the onset of the complication, dividing them into early (intraoperative), medium (early postoperative), and late (late postoperative) complications. The most prevalent complications include hemorrhage, tube obstruction, and tube displacement. Less common but notable complications encompass tracheal stenosis, granulation tissue, and tracheomalacia [1,4]. In the presented case, the patient developed granulation tissue and tracheomalacia following the tracheostomy.

Tracheomalacia is characterized by a structural abnormality of the tracheal cartilage leading to excessive collapsibility of the trachea. It involves the immaturity of cartilaginous rings, typically affecting the distal third of the trachea, resulting in weakness of the tracheal structure [5]. Tracheomalacia may manifest as localized or generalized, and it is further classified into congenital and acquired forms. Congenital tracheomalacia is attributed to the immaturity of tracheobronchial cartilage, while acquired tracheomalacia stems from the degeneration of previously normal tracheal cartilage due to inflammatory processes, extrinsic vascular compression, bronchial neoplasms, and tracheoesophageal fistulas [6].

There is currently no definitive standardized guideline for the diagnosis and evaluation of tracheomalacia. Suspicion of the condition arises from a clinical history of signs and symptoms indicative of tracheomalacia, as highlighted by Jiang et al. [7]. Accurate diagnosis relies on direct visualization and is achieved through flexible and rigid endoscopy, encompassing laryngoscopy, tracheoscopy, and bronchoscopy. Computed tomography (CT) neck and thorax has a limited role in selected cases [8].

Despite the lack of standardized criteria for establishing a diagnosis of tracheomalacia through endoscopy, there is a consensus among most surgeons that more than a 50% narrowing or collapse in the airway lumen during forced exhalation is indicative of tracheomalacia [7,8].

The pathophysiology of acquired tracheomalacia involves degeneration of previously normal tracheal cartilage, often secondary to chronic inflammation, extrinsic vascular compression, bronchial neoplasms, or tracheoesophageal fistulas. Among the various etiologies, iatrogenic causes such as prolonged endotracheal intubation with cuffed tubes and tracheostomy are the most common contributors [6].

However, recommendations suggest maintaining cuff pressures between 20 and 30 cmH_2_O in adult patients to prevent tracheal injury. Overinflation of the cuff can lead to tracheomalacia, tracheoesophageal fistula, and tracheal rupture due to injury to the tracheal mucosal blood flow [5]. Conversely, underinflation of the cuff can result in air leakage, inadequate ventilation, microaspiration, and pneumonia. In cases of tracheomalacia, it is crucial to ensure that the position of the cuff and cuff pressure are not excessive to avoid mucosal injury at the same position. Balancing cuff pressure within the recommended range is essential for optimizing patient outcomes and preventing potential complications associated with tracheal interventions [6].

Surgical treatment is reserved for the most severe cases of tracheomalacia and must be tailored to the specific type and location of tracheomalacia in each patient, based on a comprehensive diagnostic assessment protocol. Various surgical interventions have been described for tracheomalacia, including tracheostomy, tracheal resection with end-to-end anastomosis, tracheoplasty with cartilage or dura graft, prosthetic stenting, and aortic or innominate artery suspension [8].

The severity of the disease varies significantly based on the etiology and type of obstruction. Mild cases can often be managed conservatively or with the use of CPAP [7]. Fortunately, in this patient's case, the clinical outcome with conservative management was favorable. CPAP usage showed significant improvements in quality of life.

## Conclusions

In summary, this presentation highlights a case of acquired tracheomalacia arising as a complication following a tracheostomy. Elevated clinical suspicion is warranted, particularly in cases involving a history of prolonged intubation and unsuccessful extubation attempts. When confronted with such a clinical scenario, a differential diagnosis should include consideration of tracheomalacia. Confirmation of the diagnosis is achieved through the performance of flexible or rigid endoscopy, laryngoscopy, tracheoscopy, and bronchoscopy examinations. The patient responded well to CPAP, which significantly improved his symptoms and quality of life. This case underscores the importance of recognizing that the diagnosis of tracheomalacia is a multifaceted process, relying on various indicators such as patient history, clinical manifestations, endoscopic assessments, clinical suspicion, and the role of conservative management in selected patients with tracheomalacia.
